# Effects of *Radix Scrophulariae* on Hyperthyroidism Assessed by Metabonomics and Network Pharmacology

**DOI:** 10.3389/fphar.2021.727735

**Published:** 2021-09-28

**Authors:** Ning Zhang, Fang Lu, Zihui Li, Hongwei Zhao, Mu Pang, Tao Ye, Xijun Wang, Shumin Liu

**Affiliations:** ^1^ Heilongjiang University of Chinese Medicine, Harbin, China; ^2^ The First Affiliated Hospital of Guizhou University of Traditional Chinese Medicine, Guiyang, China

**Keywords:** hyperthyroidism, radix scrophulariae, mechanism of action, metabonomics, network pharmacology

## Abstract

Radix Scrophulariae (Chinese name: Xuanshen), a traditional Chinese herb, is used for the treatment of hyperthyroidism, and in this study, its mechanisms were evaluated by metabonomics and system pharmacology. To study the anti-hyperthyroidism effects of *R. Scrophulariae*, a male SD rat (180–220 g) model of hyperthyroidism induced by Euthyrox was used. Thirty rats were randomly distributed into three groups: the Model group, the *R. Scrophulariae* treatment group (RS group) and the healthy Control group. Using the UHPLC/Q-TOF-MS metabolomics approach, 44 metabolites were found to be profoundly altered in the model group, and the levels of these biomarkers were significantly decreased after treatment with *R. Scrophulariae*. Forty-four metabolites and 13 signaling pathways related to *R. Scrophulariae*, including the biosynthesis of unsaturated fatty acids, primary bile acid biosynthesis and sphingolipid metabolism, were explored, and linoleic acid metabolism and sphingolipid metabolism were identified as the most relevant metabolic pathways. In addition, the system pharmacology paradigm revealed that *R. Scrophulariae* contains 83 active ingredients and is related to 795 genes, and 804 disease genes are related to hyperthyroidism. The construction of the *R. Scrophulariaceae*-chemical composition-target-hyperthyroidism network identified a total of 112 intersection genes. The enriched gene targets were analyzed, and five pathways were found to be enriched. Among them pathways, the HIF signaling pathway had the highest enrichment score, which indicated that this pathway might be the main signaling pathway related to the treatment of hyperthyroidism by *R. Scrophulariae*.The integrated approach involving metabolomics and network pharmacology revealed that *R. Scrophulariae* might play a role in the treatment of hyperthyroidism by regulating the “IL6-APOA1-cholesterol” pathway and disturbing the HIF signaling pathway. The results demonstrate that the combination of metabolomics and network pharmacology could be used to reflect the effects of *R. Scrophulariae* on the biological network and metabolic state of hyperthyroidism and to evaluate the drug efficacy of *R.* Scrophulariaceae and its related mechanisms.

## Introduction

Hyperthyroidism, a common and frequently occurring disease of the endocrine system, is characterized by thyrotoxicosis caused by the excessive production of thyroid hormones by the thyroid gland itself (Lai et al., 2019). Hyperthyroidism is a series of syndromes due to hyperplasia or hyperfunction of thyroid tissue, excessive production and secretion of thyroid hormones, hypermetabolism and increased excitability of multiple systems (Mooradian, 2019). In recent years, the prevalence of hyperthyroidism has increased significantly as the pace of life and work continues to increase (Diab et al., 2019). Traditional Chinese medicines (TCMs) can reduce damage to the human body and improve the accuracy of the treatment. The overall factors attributed to TCMs and the human body have been comprehensively considered based on the overall concept and dialectical treatment (Yan et al., 2019).

*Radix Scrophulariae*, the dried root of *Scrophularia ningpoensis* Hemsl., which belongs to the Scrophulariaceae family, has been used in TCM for thousands of years. As detailed in the Pharmacopoeia of the People’s Republic of China (2020 Edition), the traditional functions of this species include the treatment of febrile diseases, constipation, hot eyes, pharyngalgia, diphtheria, and scrofula (National Pharmacopoeia Commission, 2015). In a previous study, a rat model of hyperthyroidism induced by Euthyrox was used as the research material. Moreover, the metabolic pathways associated with the effects of *R. Scrophulariae* extract on hyperthyroidism were explored based on the physical signs, basic metabolic rate and related indexes *in vivo* as well as the urine, serum and liver metabolomics. Finally, the results clearly show that the treatment of hyperthyroidism with *R. Scrophulariae* extract has a scientific basis (Liu et al., 2014a; Liu et al., 2014b; Liu et al., 2016; Xiao et al., 2016; Liu et al., 2017a; Liu et al., 2017b; Zhang et al., 2018; Zhang et al., 2019a; Zhang et al., 2019b).

Based on these findings, to initially explain the mechanism underlying the effects of *R. Scrophulariae* on hyperthyroidism, fecal supernatant samples of rats with hyperthyroidism that were treated with *R. Scrophulariae* were studied using an approach that involves metabolomics combined with multivariate statistical analysis techniques. Moreover, network pharmacology was used to construct a network of the relationships among *R. Scrophulariae*, its chemical composition and targets and disease. Based on the above-described metabonomics and network pharmacology approaches, the targets of *R. Scrophulariae* intervention in hyperthyroidism were systematically explained, and the results provide new ideas for the clinical treatment of hyperthyroidism and systematic research on *R. Scrophulariae*.

## Materials and Methods

### Materials

The root of *Scrophularia ningpoensis* Hemsl. is a natural medicine. *R. Scrophulariae* was acquired from the Heilongjiang Province Drug Company (Harbin, PR China), and the voucher specimen (hlj-20120623012) of the herb was authenticated by Professor Zhenyue Wang of the Department of Resources and Development of TCM at Heilongjiang University of Traditional Chinese Medicine, who found that the specimen met the standards detailed in the Pharmacopoeia of the People’s Republic of China (2020 edition, page 117) for the aqueous extract of *R. Scrophulariae* (Zhang et al., 2016).

Euthyrox^®^ (50 μg, Levothyroxine Sodium Tablets, No. H20140052) was purchased from Merck KGaA. UPLC-grade acetonitrile and formic acid were obtained from Dikma Technologies Inc. (United States), and leucine-enkephalin (No. L9133) was obtained from Sigma Aldrich (St. Louis, MO, United States).

ELISA kit for rat triiodothyronine (T3, batch Number:201605), thyroxine (T4, batch Number:201605) and norepinephrine (NE, batch Number:201605) were purchased from Nanjing Jiancheng Biotechnology Co., Ltd.,(China).

### Animals and Groups

Healthy male Sprague-Dawley rats weighing 180–220 g each were purchased from Liaoning Changsheng Biotechnology Co., Ltd. (P. R. China) (Animal Certificate No: SCXK [Liao] 2015-0001). The rats were fed a standard diet, had free access to water and were housed one per metabolic cage in controlled rooms with a temperature of 21–23°C, 40–50% humidity and a 12-h light/12-h dark cycle. This study was conducted in strict accordance with the recommendations in the Guide for the Care and Use of Laboratory Animals of the National Institutes of Health. The protocol was approved by the Committee on the Ethics of Animal Experiments of the College of Pharmacy of Heilongjiang University of Chinese Medicine (No. DWLL20151108001).

After acclimation for 1 week, 30 rats were randomly divided into three groups (n = 10 per group): the Model group, the *R. Scrophulariae* treatment group (RS group) and the healthy Control group. The rats in the Control group were fed normally every day and given 0.9% saline twice daily at an interval of 90 min for 15 consecutive days. The rats in the model group were i.g., administered 120 mg/kg/d Euthyrox suspension (dose concentration of 12 mg/ml, administration volume of 10 ml/kg) and i.g., administered saline 90 min after the Euthyrox administration; the Euthyrox suspension and saline were administered once a day for 15 days (Xiao et al., 2016). The rats in the RS group received a decoction of *R. Scrophulariae* (1350 mg of crude drug per kg, i.g.,) once daily for 15 consecutive days, and the rats in the control group received the same volume of 0.9% saline once daily for 15 days; the other interventions administered to the rats in the RS group were the same as those administered to the rats in the model group.

### Sample Collection and Preparation

All of the animals survived until the end of the treatment period, after rats were anaesthetized with 1% sodium pentobarbital (0.15 ml/100 g) via intraperitoneal injection, blood samples were collected from the abdominal aorta with pro-coagulation tubes. The serum was obtained by centrifuged at 4000 rpm for 10 min, and then T3, T4 and NE were measured by enzyme-linked immunosorbent assay.

For the metabolomic analysis, feces were collected from all the rats in each group for 24 h on the 15th day. After freeze-drying, 2.7 ml of methanol was added to 300 mg of feces, and the mixture was vortexed for 3 min, mixed by ultrasound for 15 min and centrifuged at 15,000 rpm and 4°C for 15 min. Two microliters of the supernatant was injected into the UPLC/TOF-MS instrument for analysis.

### Fecal Metabolomics

#### UHPLC/TOF-MS Analysis

The metabolomic analysis was performed using a Waters ACQUITY UPLC^®^ system coupled with a time-of-flight mass spectrometer. Chromatography was performed using an Acquity SDS ACQUITY UPLC^®^ BEH C18 column (2.1 mm × 50 mm, i. d. 1.7 µm, Waters Corp.) The column and sample temperatures were set to 40.0°C and 4.0°C, respectively. The gradient mobile phase consisted of solvent A (0.05% FA-ACN) and solvent B (0.05% FA-H_2_O). The fecal sample gradient was as follows: 0–15 min, 2.0–100% A; 15–17 min, 100% A; 17–18 min, 100–2% A; and 18–20 min, 2% A. The flow rate was 0.400 ml min^−1^. To guarantee system stability and repeatability, quality control (QC) samples were inserted every 10 samples; these samples consisted of feces obtained by mixing 100–200 ml of 10 samples from group.

The MS parameters were established as follows. The mass range was from 100 to 1500 in the full-scan mode. The desolvation temperature and source temperature were set to 350.0°C and 110.0°C, respectively. The cone and desolvation gas flow rates were maintained at 20.0 L h^−1^ and 750.0 L h^−1^, respectively. The capillary voltage was set to 1300.0 V in the positive-ion (ESI+) mode and to 1500.0 V in the negative-ion (ESI-) mode. The sample cone voltage was set to 60.0 V (ESI+) and 70.0 V (ESI-). The ion energy voltage was set to 35.0 V (ESI+) and 34.0 V (ESI-). The scan duration time and interscan delay were set to 0.200 and 0.010 s, respectively. Leucine-enkephalin was used as the lock-mass compound (556.2771 [M + H]+ and 554.2615 [M-H]^-^).

#### Data Processing and Multivariate Data Analysis

The raw data acquired by the UPLC-TOF-MS system were exported to a Progenesis QI v3.0.3 (Nonlinear Dynamics, Waters Company) workstation for peak alignment, peak picking, and deconvolution (Dionisio et al., 2019; Jia et al., 2019). The data matrix (Rt-m/z, normalized abundance, and adducts) was exported to EZinfo 3.0.3.0 software for principal component analysis (PCA), partial least squares discriminant analysis (PLS-DA) and orthogonal partial least squares discriminant analysis (OPLS-DA). We first performed a nondiscriminatory PCA analysis to detect whether each group of samples could be significantly separated (Zhang et al., 2019c). If the identified metabolites were differential components, they were removed from the original data and analyzed by PCA. After these analyses, 2D or 3D-PCA score plots reflected the clustering degree of each group. To analyze the fecal metabolic profiles of the experimental and control groups, OPLS-DA score plots were constructed to obtain VIP plots, S-plots and loading plots (Yi et al., 2019). The variables located farther from the origin in these plots contributed significantly. Using the criteria *p* ≤ 0.05 and variable importance in the projection (VIP) > 1, potential biomarkers were selected and compared using HMDB (http://www.hmdb.ca/) and Progenesis MetaScope. Metabolic pathway analysis was performed using KEGG (http://www.kegg.jp/), and Cytoscape 3.7.1 software was used to visualize the obtained network diagrams of compounds and pathways.

### Network Pharmacology Study

First, the literature on *R. Scrophulariae* obtained through a search of various databases, such as TCMSP (http://lsp.nwu.edu.cn/tcmspsearch.php), TCM-ID (http://bidd.nus.edu.sg/group/TCMsite/Default.aspx), BATMAN-TCM (http://bionet.cpsb.org/batman-tcm/), and TCMGeneDIT (http://tcm.lifescience.ntu.edu.tw/index.html), was extensively studied to retrieve all chemical ingredients and their protein targets. Second, the molecular targets and genes associated with hyperthyroidism were ascertained from databases such as MalaCards (https://www.malacards.org), GenBank (https://www.ncbi.nlm.nih.gov/genbank/), GeneCards (https://www.genecards.org) and OMIM (https://www.omim.org).

Third, using the UniProt database, the target names obtained from the TCMSP, TCM-ID,BATMAN-TCM,TCMGeneDIT, MalaCards,GenBank, GeneCards and OMIM databases were inputted, and the corresponding gene names were obtained; in addition, the protein names were corrected. The gene names included in Swiss-Prot and that were highly matched were preferred. The corrected genes of *R. Scrophulariae* that intersected with hyperthyroidism were identified. Fourth, using the DAVID (http://david.nifcrf.gov/) database, the obtained intersecting genes were subjected to a GO annotation analysis (Gene-Ontology) and a KEGG pathway analysis (Kyoto Encyclopedia of Genes and Genomes). The intersection genes could be directly mapped onto the pathways. Finally, according to the target prediction results obtained for the above-mentioned chemical constituents of *R. Scrophularia*, a network model of the chemical constituents, intersections, and target pathways was constructed using Cytoscape 3.7.1. If a target is a potential target of a compound, it is connected by an edge. If a target participates in a certain pathway, the target is also connected to the action pathway by an edge. Through the construction of the network, the characteristics of multiple components, multiple targets, and multiple pathways associated with the treatment of hyperthyroidism by *R. Scrophularia* were revealed (Guo et al., 2020).

### Integrated Analysis Involving Metabonomics and Network Pharmacology

The gene targets corresponding to the potential biomarkers were searched in the HMDB database. In the String database, the pattern of multiple proteins was selected, and an interaction network diagram of the gene targets of potential biomarkers and related signaling pathways associated with the main intervention of *R. Scrophularia* was drawn.

## Results

### The Levels of T3, T4 and NE in the Rat Serum

The levels of T3 and T4 were significantly reduced in R. Scrophularia group relative to that in model group, indicating that Radix Scrophulariae extract can play a therapeutic role by improving the endocrine system of Hyperthyroidism in Rats. Likewise, Radix Scrophulariae extract can also reduce NE content in Hyperthyroidism Rats (*p* < 0.05) (Table 1), indirectly indicating that Radix Scrophulariae extract has a protective effect on autonomic nervous system of Hyperthyroidism Rats Induced by Euthyrox.

**TABLE 1:** Effects of Radix Scrophulariae extract on T3, T4 and NE in hyperthyroidism model rats ( $\bar{x}$ ± s, n = 10).

Groups	T3 (ng/mL)	T4 (ng/mL)	NE (nmol)
Control group	0.63 ± 0.13	8.30 ± 0.95	2.89 ± 0.38
Model group	0.78 ± 0.13^▲^	10.96 ± 1.70^▲▲^	3.30 ± 0.30^▲^
R.Scrophularia group	0.64 ± 0.07^★^	8.66 ± 1.42^★^	2.87 ± 0.42^★^

compared with the control group, ▲ *p* < 0.05, ▲▲ *p* < 0.01; Compared with the model group, ★ *p* < 0.05, ★★ *p* < 0.01.

### Fecal Metabolic Profiling

#### Metabolic Fingerprint

**FIGURE 1:**
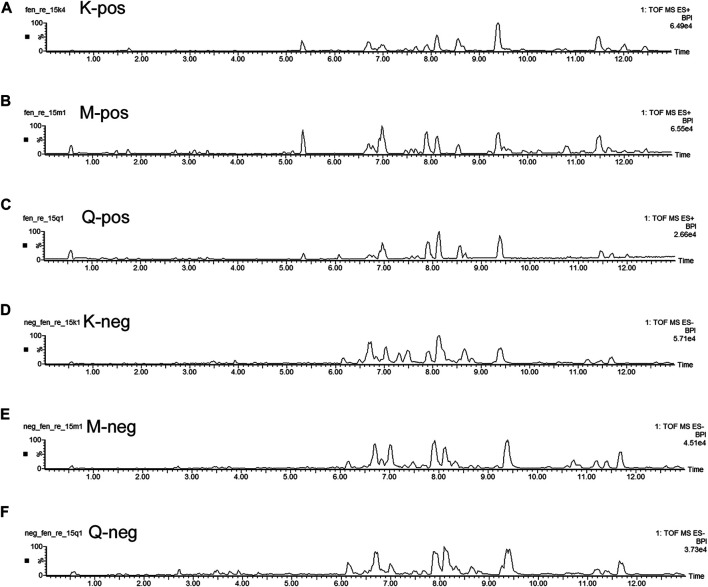
BPI chromatograms of the *R. Scrophulariae* extract, model and control groups. K represents the Control group, M represents the Model group, and Q represents the *R. Scrophulariae* group.

The basal peak intensity (BPI) chromatograms of the fecal supernatants of the Control, Model and RS groups are shown in Figure 1. Due to the packing of small particles in the UPLC chromatography column, small molecular metabolites could be well separated in just 13 min.

This study found a significant difference in the metabolic spectra of fecal supernatants between the feces of the Control rats and those of the Model rats, which indicated that the rats belonging to the Model group had unique characteristics of fecal metabolites. This finding suggested that a changes in single substances did not serve as biomarkers of *R. Scrophularia* and Euthyrox; instead, a variety of metabolites formed a biomarker group for fecal supernatants.

**FIGURE 2:**
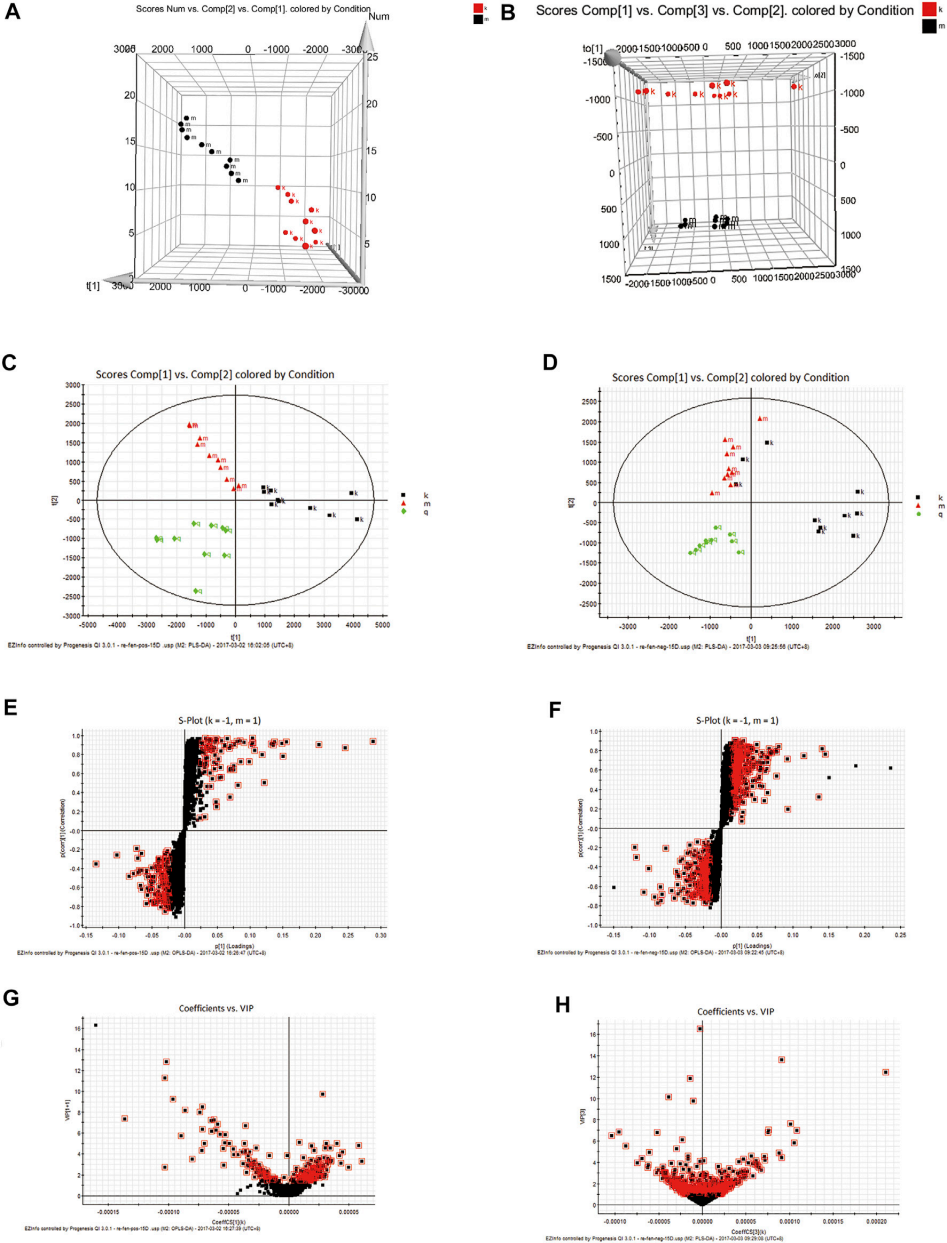
3D-PCA plot based on data from the Control and Model groups obtained in the positive **(A)**—and negative **(B)**—ion modes. PCA plot based on data from the Control, Model and *Radix Scrophulariae* groups obtained in the positive **(C)**—and negative **(D)**—ion modes (n = 10). S-plots of fecal samples from the Control and Model groups obtained in both the positive **(E)**- and negative **(F)**—ion modes. VIP plots of fecal samples from the Control and Model groups obtained in both the positive **(G)**—and negative **(H)**-ion modes.

EZinfo was used to perform a 3D-PCA analysis of the Control and Model groups (Figures 2A,B). Good separation was achieved with both groups. The results showed a significant difference in the content of endogenous metabolites between the Control and Model groups.44 significant differential metabolites produced in the faecal after the intervention of R. Scrophulariae in model rats (Table 2). A PLS-DA (Figures 2C,D) analysis was performed using the data from the Control, Model and RS groups. The PLS-DA clustering score chart revealed good separation for all three groups, and the RS group was located between the Model and Control groups. The above-described results show that the abnormal metabolism was improved after the administration of *R. Scrophulariae*, which indicated that *R. Scrophulariae* exerts an improved therapeutic effect. The results from the metabonomics analysis are more intuitive, clear and comprehensive and thus reflect the advantages of model induction and *R. Scrophulariae* intervention on hyperthyroidism.

**TABLE 2:** Significant differential metabolites produced in the faecal after the intervention of *R. Scrophulariae* in model rats.

No	Compound	VIP	Ion mode	HMDB ID	Formula	Score	Description	m/z	Rt (min)	Anova (p)	q Value	Max fold change	M Vs K	Q vs M
F1	7.02_408.2907n	6.6905	pos	HMDB00415	C24H40O5	53.8	3a,6b,7b-Trihydroxy-5b-cholanoic acid	817.5887	7.0193	0.0229	0.0063	2.3117	up	down
F2	7.02_408.2907n	6.6905	pos	HMDB00619	C24H40O5	53.8	Cholic acid	817.5887	7.0193	0.0229	0.0063	2.3117	up	down
F3	7.02_408.2907n	6.6905	pos	HMDB00760	C24H40O5	53.8	Hyocholic acid	817.5887	7.0193	0.0229	0.0063	2.3117	up	down
F4	10.86_280.2418n	3.2749	pos	HMDB00673	C18H32O2	57.7	Linoleic acid	281.2490	10.8574	0.0051	0.0019	3.4695	down	up
F5	10.86_280.2418n	3.2749	pos	HMDB06270	C18H32O2	57.7	Linoelaidic acid	281.2490	10.8574	0.0051	0.0019	3.4695	down	up
F6	10.21_299.2823n	3.2181	pos	HMDB00252	C18H37NO2	41.4	Sphingosine	300.2895	10.2099	0.0165	0.0048	2.1302	down	up
F7	10.21_299.2823n	3.2181	pos	HMDB01480	C18H37NO2	41.4	3-Dehydrosphinganine	300.2895	10.2099	0.0165	0.0048	2.1302	down	up
F8	10.21_299.2823n	3.2181	pos	HMDB02100	C18H37NO2	41.0	Palmitoylethanolamide	300.2895	10.2099	0.0165	0.0048	2.1302	down	up
F9	8.14_390.2806n	2.2591	pos	HMDB00467	C24H38O4	52.7	Nutriacholic acid	373.2773	8.1361	0.0066	0.0023	2.1578	down	up
F10	1.28_131.0949n	3.1782	pos	HMDB00557	C6H13NO2	39.3	l-Alloisoleucine	132.1022	1.2811	0.0026	0.0011	2.2741	up	down
F11	1.28_131.0949n	3.1782	pos	HMDB01645	C6H13NO2	39.3	l-Norleucine	132.1022	1.2811	0.0026	0.0011	2.2741	up	down
F12	8.33_416.3171m/z	1.7504	pos	HMDB00698	C26H43NO4	42.4	Lithocholic acid glycine conjugate	416.3171	8.3278	0.0001	0.0001	3.1697	down	up
F13	11.09_279.1606m/z	1.4903	pos	HMDB13243	C15H22N2O3	33.8	Leucyl-phenylalanine	279.1606	11.0926	0.0001	0.0001	6.1519	down	up
F14	11.09_279.1606m/z	1.4903	pos	HMDB28757	C10H18N2O5	34.6	Aspartyl-Leucine	279.1606	11.0926	0.0001	0.0001	6.1519	down	up
F15	11.09_279.1606m/z	1.4903	pos	HMDB28852	C13H15N3O3	31.1	Glycyl-Tryptophan	279.1606	11.0926	0.0001	0.0001	6.1519	down	up
F16	11.09_279.1606m/z	1.4903	pos	HMDB28903	C10H18N2O5	34.6	Isoleucyl-Aspartate	279.1606	11.0926	0.0001	0.0001	6.1519	down	up
F17	11.09_279.1606m/z	1.4903	pos	HMDB28914	C15H22N2O3	33.8	Isoleucyl-Phenylalanine	279.1606	11.0926	0.0001	0.0001	6.1519	down	up
F18	11.09_279.1606m/z	1.4903	pos	HMDB28998	C15H22N2O3	33.8	Phenylalanyl-Isoleucine	279.1606	11.0926	0.0001	0.0001	6.1519	down	up
F19	11.09_279.1606m/z	1.4903	pos	HMDB29083	C13H15N3O3	31.1	Tryptophyl-Glycine	279.1606	11.0926	0.0001	0.0001	6.1519	down	up
F20	11.09_279.1606m/z	1.4903	pos	HMDB31342	C7H12O2	35.0	Cyclohexanecarboxylic acid	279.1606	11.0926	0.0001	0.0001	6.1519	down	up
F21	11.09_279.1606m/z	1.4903	pos	HMDB31476	C7H12O2	35.0	2,3-Heptanedione	279.1606	11.0926	0.0001	0.0001	6.1519	down	up
F22	11.09_279.1606m/z	1.4903	pos	HMDB59717	C10H18N2O5	34.6	Glutamylvaline	279.1606	11.0926	0.0001	0.0001	6.1519	down	up
F23	13.59_371.3679m/z	2.4270	pos	HMDB00577	C27H48O	40.0	5beta-Coprostanol	371.3679	13.5888	0.0065	0.0023	5.8141	down	up
F24	13.59_371.3679m/z	2.4270	pos	HMDB00908	C27H48O	40.0	5alpha-Cholestanol	371.3679	13.5888	0.0065	0.0023	5.8141	down	up
F25	13.59_371.3679m/z	2.4270	pos	HMDB01569	C27H48O	40.0	Epi-coprostanol	371.3679	13.5888	0.0065	0.0023	5.8141	down	up
F26	13.87_409.3476m/z	1.7883	pos	HMDB00067	C27H46O	53.8	Cholesterol	409.3476	13.8666	0.0002	0.0002	2.8693	down	up
F27	14.82_128.1441m/z	4.4377	pos	HMDB01257	C7H19N3	30.3	Spermidine	128.1441	14.8245	0.0020	0.0009	1.8835	up	down
F28	14.82_128.1441m/z	4.4377	pos	HMDB29600	C6H14	37.4	Hexane	128.1441	14.8245	0.0020	0.0009	1.8835	up	down
F29	8.33_408.3961m/z	2.3692	pos	HMDB02368	C24H46O2	33.3	Nervonic acid	408.3961	8.3278	0.0000	0.0000	16.0056	down	up
F30	7.40_353.2343m/z	2.8171	pos	HMDB06528	C22H34O2	41.6	Docosapentaenoic acid	353.2343	7.4030	0.0000	0.0000	6.2636	down	up
F31	7.40_353.2343m/z	2.8171	pos	HMDB60113	C22H34O2	41.6	Docosa-4,7,10,13,16-pentaenoic acid	353.2343	7.4030	0.0000	0.0000	6.2636	down	up
F32	1.66_196.0181m/z	5.2704	pos	HMDB00132	C5H5N5O	34.5	Guanine	196.0181	1.6635	0.0016	0.0007	4.4662	up	down
F33	3.59_228.1354n	1.6623	neg	HMDB11175	C11H20N2O3	31.8	L-leucyl-l-proline	227.1281	3.5925	0.0213	0.0156	1.2140	down	up
F34	3.59_228.1354n	1.6623	neg	HMDB28937	C11H20N2O3	31.8	Leucyl-Proline	227.1281	3.5925	0.0213	0.0156	1.2140	down	up
F35	14.21_256.2429n	1.7521	neg	HMDB00220	C16H32O2	37.5	Palmitic acid	301.2411	14.2145	0.0009	0.0012	1.6847	up	down
F36	14.21_256.2429n	1.7521	neg	HMDB31068	C16H32O2	37.5	Isopalmitic acid	301.2411	14.2145	0.0009	0.0012	1.6847	up	down
F37	14.21_256.2429n	1.7521	neg	HMDB60083	C16H32O2	37.5	Hexadecanoate (n-C16:0)	301.2411	14.2145	0.0009	0.0012	1.6847	up	down
F38	14.35_449.1612m/z	3.7201	neg	HMDB31472	C6H14S2	33.0	Dipropyl disulfide	449.1612	14.3480	0.0002	0.0003	3.1637	up	down
F39	9.44_527.2618m/z	3.2600	neg	HMDB29008	C14H20N2O3	47.8	Phenylalanyl-Valine	527.2618	9.4367	0.0000	0.0000	3.1423	up	down
F40	9.44_527.2618m/z	3.2600	neg	HMDB29134	C14H20N2O3	47.8	Valyl-Phenylalanine	527.2618	9.4367	0.0000	0.0000	3.1423	up	down
F41	6.69_470.2773m/z	1.7244	neg	HMDB00631	C26H43NO5	33.3	Deoxycholic acid glycine conjugate	470.2773	6.6886	0.0036	0.0036	1.6354	down	up
F42	6.69_470.2773m/z	1.7244	neg	HMDB00637	C26H43NO5	33.3	Chenodeoxycholic acid glycine conjugate	470.2773	6.6886	0.0036	0.0036	1.6354	down	up
F43	6.69_470.2773m/z	1.7244	neg	HMDB00708	C26H43NO5	33.3	Glycoursodeoxycholic acid	470.2773	6.6886	0.0036	0.0036	1.6354	down	up
F44	8.68_523.2289m/z	2.6177	neg	HMDB11177	C14H18N2O3	36.7	L-phenylalanyl-l-proline	523.2289	8.6772	0.0000	0.0000	3.4567	down	up

F represents feces. K represents control group, M represents model group, Q represents R. Scrophulariae group.

#### Identification of Potential Biomarkers

In the PLS-DA analysis, each point in the PLS-DA score plot of the Control, Model and RS groups (Figures 2E,F) represents a variable. The VIP was measured by its value, and the variable was screened according to the VIP value (Furukawa et al., 2019). The variables with VIP>1 (Figures 2G,H) and that exhibited statistical significance were considered to significantly contribute to the model. In the direction of difference among the three groups, the farther the distance from the center, the greater the contribution to the difference, and the more likely that a metabolite is a potential characteristic metabolite.

**FIGURE 3:**
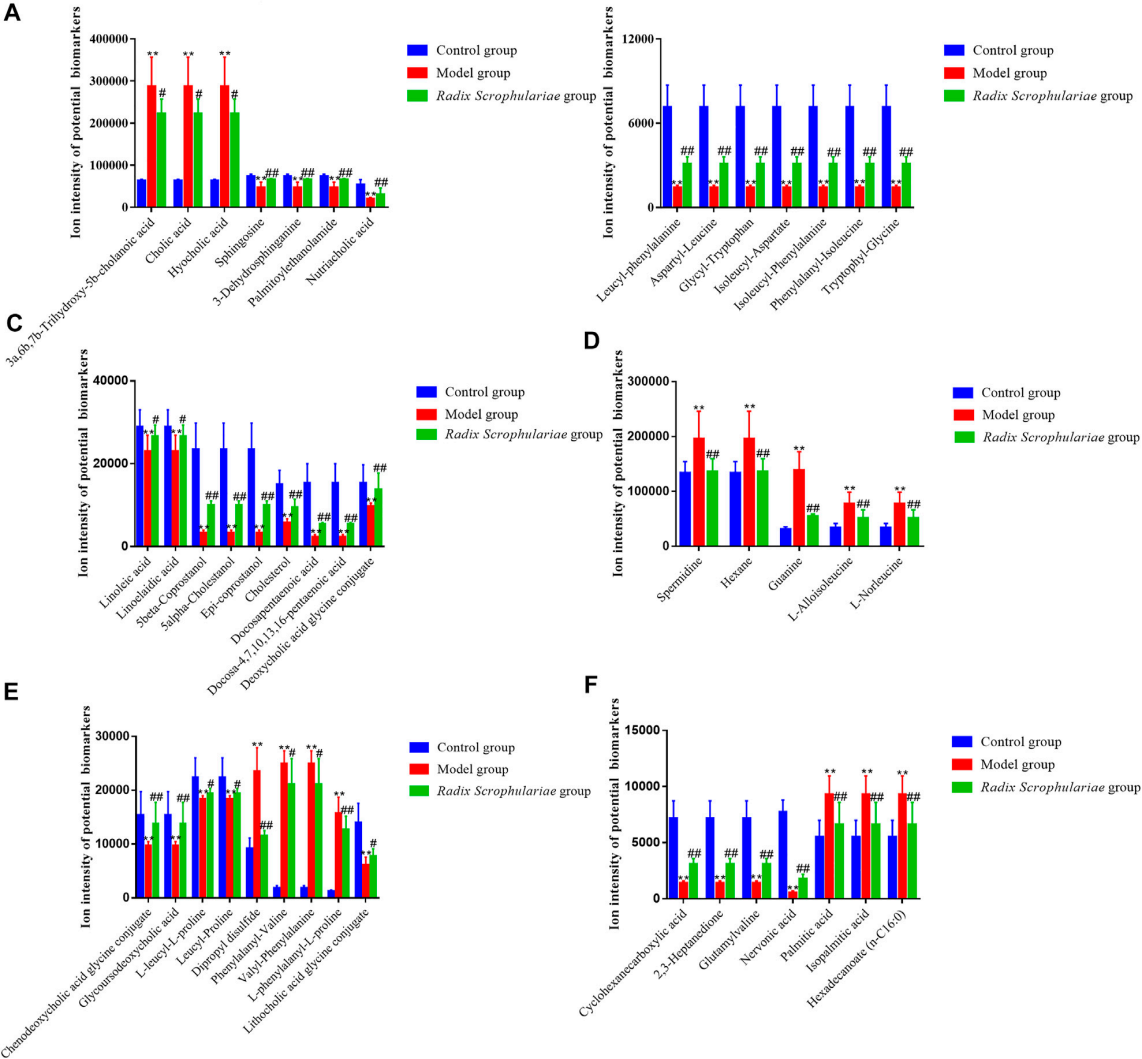
Ion intensities of potential biomarkers in fecal samples from the different groups **(A–F)**. The data are presented as the means ± SEs from each group (n = 10 in each group). *Altered trend compared with the Control group, *p* < 0.05; **altered trend compared with the control group, *p* < 0.01.

**FIGURE 4:**
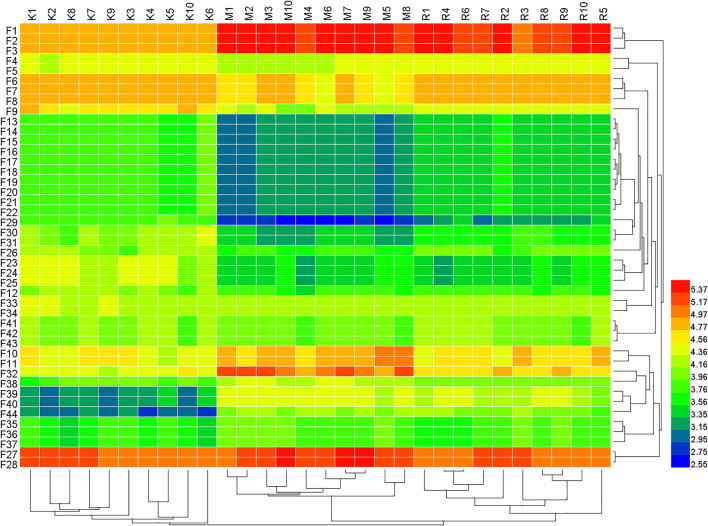
Heat map of the differences in the potential biomarkers of the Control, Model and *R. Scrophulariae* groups obtained in the positive- and negative-ion modes. F represents feces. K represents control group, M represents model group, R represents R. Scrophulariae group.

Based on tandem mass spectrometry data, potential biomarkers were retrieved and confirmed in the HMDB (http://www.hmdb.ca/), METLIN (http://www.metlin.scipps.edu/), KEGG (http://www.genome.jp/kegg/) and SMPD (http://www.smpdb.ca/) databases. A total of 44 potential biomarkers were identified through comparisons with the literature (Table 3 and Figure 3, 4). 3a,6b, 7b-Trihydroxy-5b-cholanoic acid, cholic acid, hyocholic acid, l-alloisoleucine, l-norleucine, spermidine, hexane, guanine, palmitic acid, isopalmitic acid, hexadecanoate (n-c16:0), dipropyl disulfide,phenylalanyl-valine, and valyl-phenylalanine were upregulated in the fecal supernatants of the Model group compared the supernatants of the Control group, whereas linoleic acid, linoelaidic acid, sphingosine,3-dehydrosphinganine, palmitoylethanolamide, nutriacholic acid, lithocholic acid glycine conjugate, leucyl-phenylalanine,aspartyl-leucine, glycyl-tryptophan,isoleucyl-aspartate,isoleucyl-phenylalanine,phenylalanyl-isoleucine, tryptophylglycine, cyclohexanecarboxylic acid,2,3-heptanedione,glutamylvaline, 5beta-coprostanol, 5alpha-cholestanol,epi-coprostanol,cholesterol, nervonic acid, docosapentaenoic acid,docosa-4,7,10,13,16-pentaenoic acid,l-leucyl-l-proline, leucyl-proline, deoxycholic acid glycine conjugate, chenodeoxycholic acid glycine conjugate, glycoursodeoxycholic acid, and l-phenylalanyl-l-proline were downregulated. After treatment with *R. Scrophulariae*, the levels of these biomarkers changed to the levels found in the Control group, which indicated that *R. Scrophulariae* extract can regulate the metabolism of hyperthyroid rats.

**TABLE 3:** Metabolic pathway analysis based on MetPA.

No	Pathway name	Class	Total	Expected	Hits	Raw p	-Log(P)	Holm adjust	FDR	Impact
1	Biosynthesis of unsaturated fatty acids	Lipid metabolism	42	0.6291	4	0.0029	5.8503	0.2332	0.2332	0.0000
2	Primary bile acid biosynthesis	Lipid metabolism	46	0.6890	3	0.0291	3.5383	1.0000	1.0000	0.0667
3	Sphingolipid metabolism	Lipid metabolism	21	0.3146	2	0.0378	3.2749	1.0000	1.0000	0.1353
4	Linoleic acid metabolism	Lipid metabolism	5	0.0749	1	0.0728	2.6203	1.0000	1.0000	1.0000
5	beta-Alanine metabolism	Metabolism of other amino acids	19	0.2846	1	0.2507	1.3835	1.0000	1.0000	0.0000
6	Glutathione metabolism	Metabolism of other amino acids	26	0.3894	1	0.3270	1.1179	1.0000	1.0000	0.0115
7	Fatty acid elongation in mitochondria	Lipid metabolism; Fatty acid metabolism	27	0.4044	1	0.3372	1.0870	1.0000	1.0000	0.0000
8	Steroid biosynthesis	Lipid metabolism	35	0.5243	1	0.4142	0.8814	1.0000	1.0000	0.0539
9	Fatty acid metabolism	Fatty acid metabolism	39	0.5842	1	0.4494	0.7998	1.0000	1.0000	0.0000
10	Fatty acid biosynthesis	Lipid metabolism	43	0.6441	1	0.4826	0.7286	1.0000	1.0000	0.0000
11	Arginine and proline metabolism	Amino acid metabolism	44	0.6591	1	0.4906	0.7121	1.0000	1.0000	0.0341
12	Purine metabolism	Nucleotide metabolism	68	1.0185	1	0.6507	0.4297	1.0000	1.0000	0.0103
13	Steroid hormone biosynthesis	Lipid metabolism	70	1.0485	1	0.6616	0.4131	1.0000	1.0000	0.0175

#### Metabolic Pathway Analysis

The biomarkers shown in Table 4 were inputted into MetPA (http://metpa.metabolomics.ca./MetPA/faces/Home.jsp) to construct and analyze the related metabolic pathways. The critical value of the metabolic pathway impact value was set to 0.10. If the value was higher than this threshold, the pathway would be selected as a potential target path.

**TABLE 4:** Chemical composition of *R.*Scrophulariaceae in different databases.

No	Molecule name	From	No	Molecule name	From	No	Molecule name	From
C1	vanillic acid	TCMSP	C32	scropolioside D	TCMSP	C63	nonanoic acid	TCM-ID
C2	cis-Zimtsaeure	TCMSP	C33	scropolioside D_qt	TCMSP	C64	paeonol	TCM-ID
C3	hydroxytyrosol	TCMSP	C34	harpagoside	TCMSP	C65	para-hydroxy-acetophenone	TCM-ID
C4	acteoside	TCMSP	C35	harpagoside_qt	TCMSP	C66	p-methoxycinnamic acid	TCM-ID
C5	decaffeoylacteoside	TCMSP	C36	catapol	TCMSP	C67	scrohularin	TCM-ID
C6	p-MCA	TCMSP	C37	geniposide	TCMSP	C68	tetradecanoic acid	TCM-ID
C7	paeoniflorin_qt	TCMSP	C38	geniposide_qt	TCMSP	C69	(-)-Nissolin	BATMAN-TCM
C8	sugiol	TCMSP	C39	5-(methylthio)valeronitrile	TCMSP	C70	Harpagoside	BATMAN-TCM
C9	caffeic acid	TCMSP	C40	6′-O-cinnamoylharpagirle	TCMSP	C71	Ningposide B	BATMAN-TCM
C10	cinnamic acid	TCMSP	C41	6′-O-cinnamoylharpagirle_qt 2	TCMSP	C72	3-O-Acetyl-2-O-(P-Hydroxycinnamoyl)-Alpha-l-Rhamnose	BATMAN-TCM
C11	catapol_qt	TCMSP	C42	6-O-methylcatalpol	TCMSP	C73	l-Asparagine	BATMAN-TCM
C12	5-(2-hydroxyethyl)-2-methoxyphenol	TCMSP	C43	6-O-methylcatalpol_qt	TCMSP	C74	Harpagide	BATMAN-TCM
C13	succinic acid	TCMSP	C44	harpagide	TCMSP	C75	8-O-(2-Hydroxycinnamoyl)Harpagide	BATMAN-TCM
C14	lupeol	TCMSP	C45	7-hydroxy-9-hydroxymethyl-3-oxo-	TCMSP	C76	6-O-Methyl Catalpol	BATMAN-TCM
C15	Sitogluside	TCMSP	C46	cistanoside D	TCMSP	C77	Methylchavicol	BATMAN-TCM
C16	beta-sitosterol	TCMSP	C47	Cedrol	TCMSP	C78	6′-O-Acethylharpagoside	BATMAN-TCM
C17	sitosterol	TCMSP	C48	24-dihydroxy acetophenone	TCM-ID	C79	8-O-Feruloylharpagide	BATMAN-TCM
C18	FER	TCMSP	C49	2-hydroxy-5-methoxyacetophenone	TCM-ID	C80	Ningposide A	BATMAN-TCM
C19	aucubin	TCMSP	C50	2-methyl naphthalene	TCM-ID	C81	P-Methoxycinnamic Acid	BATMAN-TCM
C20	FERULIC ACID (CIS)	TCMSP	C51	3-methoxyphenol	TCM-ID	C82	8-(o-methyl-p-coumaroyl)-harpagide	TCMGeneDIT
C21	Crystal VI	TCMSP	C52	4-hydroxy-35-dimethoxyacetophenone	TCM-ID	C83	Asparagine	TCMGeneDIT
C22	ursolic acid	TCMSP	C53	4-methyl-2-methoxyphenol	TCM-ID	C84	Carotene	TCMGeneDIT
C23	aucubigenin	TCMSP	C54	8- (o-methyl-p-coumaroyl) -harpagide	TCM-ID	C85	Linoleic acid	TCMGeneDIT
C24	oleic acid	TCMSP	C55	acetophenone	TCM-ID	C86	Oleic acid	TCMGeneDIT
C25	paeonioflorin	TCMSP	C56	anthracene	TCM-ID	C87	Stearic acid	TCMGeneDIT
C26	Homovanillyl alcohol	TCMSP	C57	caprylic acid	TCM-ID	C88	harpagoside	TCMGeneDIT
C27	HMF	TCMSP	C58	ethyl benzene	TCM-ID	C89	methoxycinnamic acid	TCMGeneDIT
C28	angroside C	TCMSP	C59	harpagoside	TCM-ID	C90	p-methoxycinnamic acid	TCMGeneDIT
C29	scropolioside A	TCMSP	C60	hexadecanoic acid	TCM-ID	C91	scrohularin	TCMGeneDIT
C30	scropolioside A_qt	TCMSP	C61	hexanoic acid	TCM-ID			
C31	14-deoxy-12(R)-sulfoandrographolide	TCMSP	C62	methoxycinnamic acid	TCM-ID			

**FIGURE 5:**
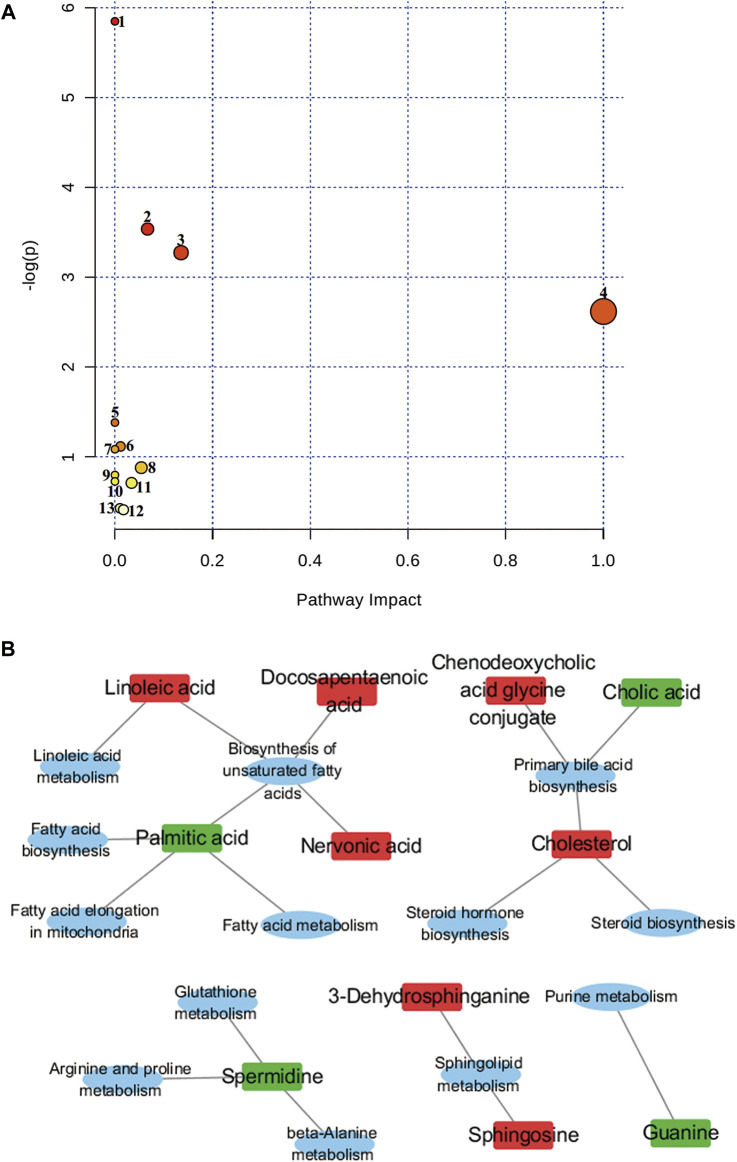
Topological mapping of potential biomarkers based on METPA analysis **(A)**. (1) Biosynthesis of unsaturated fatty acids, (2) primary bile acid biosynthesis, (3) sphingolipid metabolism, (4) linoleic acid metabolism, (5) beta-alanine metabolism, (6) glutathione metabolism, (7) fatty acid elongation in mitochondria, (8) steroid biosynthesis, (9) fatty acid metabolism (10) fatty acid biosynthesis (11) arginine and proline metabolism (12) purine metabolism and (13) steroid hormone biosynthesis. Relationship between metabolic pathways and related metabolites of *R. Scrophulariae* involved in the treatment of hyperthyroidism rats **(B)**. The elliptical nodes represent pathways, and the rectangular nodes represent metabolites (red: upregulation, green: downregulation).

As shown in Figure 5, nine of the metabolic pathways associated with Euthyrox-induced metabolic disorders in hyperthyroid rats were attributed to lipid metabolism, whereas two, one and one of the pathways were respectively related to other amino acid metabolism, nucleic acid metabolism, and amino acid metabolism, particularly the linoleic acid and sphingolipid metabolism pathways in lipid metabolism. In the RS group, the metabolite contents were adjusted back to the baseline levels, and the disorder in the metabolic pathways was improved. The metabolic-level analysis verified that *R.* Scrophulariaceae exerts a good therapeutic effect on the hyperthyroidism model induced by Euthyrox.

Of the 44 markers identified, nervonic acid, docosapentaenoic acid, palmitic acid and linoleic acid are mainly involved in unsaturated fatty acid biosynthesis, and cholesterol, chenodeoxycholic acid glycine conjugate and cholic acid are mainly involved in primary bile acid biosynthesis.3-Dehydrosphinganine and sphingosine mainly participate in sphingolipid metabolism, and spermidine is mainly involved in β-alanine metabolism, glutathione metabolism, and arginine and proline metabolism. Linoleic acid is mainly involved in linoleic acid metabolism. Palmitic acid is mostly involved in mitochondrial fatty acid elongation, fatty acid metabolism and fatty acid biosynthesis, and cholesterol is mainly involved in steroid biosynthesis and steroid hormone biosynthesis. Guanine is mainly involved in purine metabolism. The results showed that hyperthyroidism can induce a series of complex metabolic pathway disorders, such as lipid metabolism, amino acid metabolism and nucleic acid metabolism. *R.* Scrophulariaceae can play a therapeutic role by regulating the metabolic network and metabolic pathways involved in Euthyrox-induced hyperthyroidism in rats.

### Results of Network Pharmacology

#### Collection of Chemical Components and Target Genes

**FIGURE 6:**
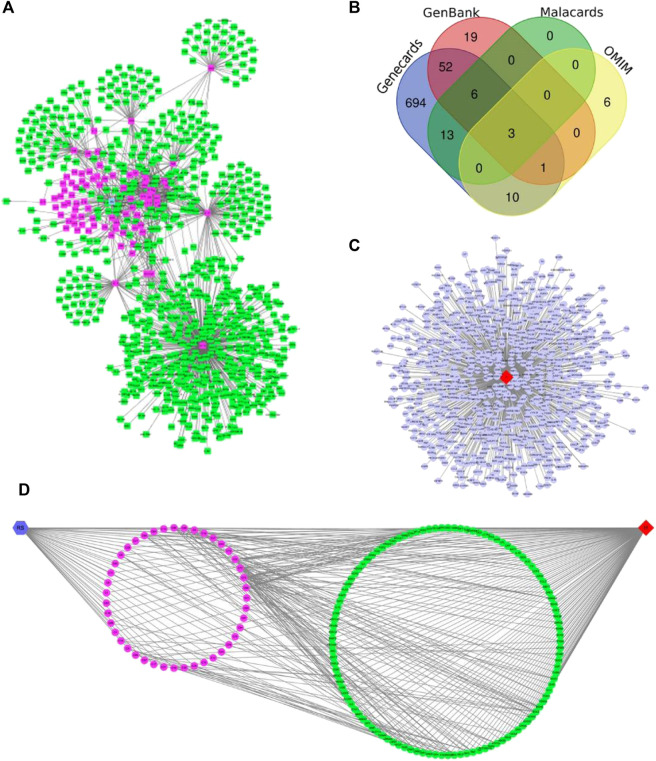
**(A)** Chemical constituents and targets of *R. Scrophulariae* (red color: components; green nodes: targets). **(B)** Venn diagram of hyperthyroidism-related targets. **(C)** Hyperthyroidism-related targets. The red diamond nodes represent hyperthyroidism, and the blue dot nodes represent targets related to hyperthyroidism. **(D)** Relationship among *R. Scrophulariae*, its chemical composition and targets and disease. The blue hexagon node represents *R. Scrophulariae*, the pink circle nodes represent the chemical compositions, the green circle nodes represents the common targets, and the red diamond represents the disease.

Forty-seven,21,13 and 10 chemical constituents of *R. Scrophulariae* were retrieved from TCMSP, TCM-ID, BATMAN-TCM, and TCMGeneDIT, respectively, and 83 chemical constituents of *R. Scrophulariae* were obtained by deleting the intersection. These constituents were divided into 11 categories:iridoids, phenylpropanoid glycosides, phytosterols, organic acids, flavonoids, triterpene saponins, volatile oils, sugars, amino acids, alkaloids and monoterpene and diterpene components and other compounds. The chemical composition of *R. Scrophulariae* matched 795 genes, such as PTGS2, MAOB, and MAOA (Table 5 and Figure 6A).

**TABLE 5:** Metabolic pathway information.

No	GO term	Count	Gene ratio%	*p* value	Associated genes
1	Malaria	11	22.45	3.17E-11	CXCL8, ICAM1, IL10, IL1B, IL6, PECAM1, SELE, SELP, TGFB1, TLR4, TNF
2	Amyotrophic lateral sclerosis (ALS)	10	19.61	1.10E-09	BCL2, BCL2L1, CAT, GRIN1, GRIN2A, GRIN2B, NOS1, SOD1, TNF, TP53
3	Type I diabetes mellitus	7	16.28	1.49E-06	FASLG, GAD1, GAD2, IL1B, IL2, INS, TNF
4	Aldosterone-regulated sodium reabsorption	5	13.51	1.29E-04	ATP1A1, ATP1A2, INS, INSR, NR3C2
5	HIF-1 signaling pathway	13	13.00	6.56E-10	AKT1, BCL2, EP300, ERBB2, HIF1A, IL6, INS, INSR, NOS3, SERPINE1, SLC2A1, TLR4, VEGFA
6	Chagas disease (American trypanosomiasis)	13	12.62	9.55E-10	AKT1, CCL3, CXCL8, FASLG, FOS, IL10, IL1B, IL2, IL6, SERPINE1, TGFB1, TLR4, TNF
7	Thyroid hormone signaling pathway	12	10.34	4.35E-08	AKT1, ATP1A1, ATP1A2, EP300, ESR1, HIF1A, NCOA1, RXRB, SLC2A1, SRC, STAT1, TP53
8	Prolactin signaling pathway	7	10.00	4.12E-05	AKT1, ESR1, FOS, INS, SRC, STAT1, TH
9	Prostate cancer	9	9.28	5.84E-06	AKT1, BCL2, EP300, ERBB2, INS, MMP3, PLAT, PTEN, TP53
10	Hepatitis B	14	8.59	3.34E-08	AKT1, BCL2, CCNA2, CXCL8, EP300, FASLG, FOS, IL6, SRC, STAT1, TGFB1, TLR4, TNF, TP53
11	cGMP-PKG signaling pathway	13	7.83	3.18E-07	ADORA1, ADRA1B, ADRB2, AKT1, ATP1A1, ATP1A2, INS, INSR, NOS3, NPPC, PDE2A, PDE3B, SLC25A4
12	cAMP signaling pathway	16	7.55	2.04E-08	ADORA1, ADRB2, AKT1, ATP1A1, ATP1A2, CHRM2, EP300, FOS, GRIN1, GRIN2A, GRIN2B, HCN2, PDE10A, PDE3B, PPARA, SOX9
13	Pathways in cancer	27	5.09	1.01E-09	AKT1, BCL2, BCL2L1, CXCL8, EP300, ERBB2, ESR1, F2, FASLG, FGF10, FGF2, FGF4, FOS, HIF1A, IGF2, IL2, IL6, MMP1, NCOA1, PTEN, RXRB, SLC2A1, STAT1, TGFB1, TP53, TXNRD1, VEGFA

#### Target Information Related to Hyperthyroidism

From the MalaCards, GenBank, GeneCards, and OMIM databases, 22, 81, 779, and 20 hyperthyroidism-related genes were obtained, respectively, and after the intersection genes were deleted, a total of 804 hyperthyroidism-related genes were identified (Figures 6B,C).

#### *R. Scrophulariae*-Component-Target-Disease Network Construction

Using Merge in Cytoscape, we obtained the *R. Scrophulariae*-component- target-disease network diagram (Figure 6D). A total of 112 genes related to *R. Scrophulariae* and hyperthyroidism were obtained, and these included PTEN, EP300, and CCNB1.

#### Gene Biomolecule Functional Annotation and Pathway Enrichment Analysis

**FIGURE 7:**
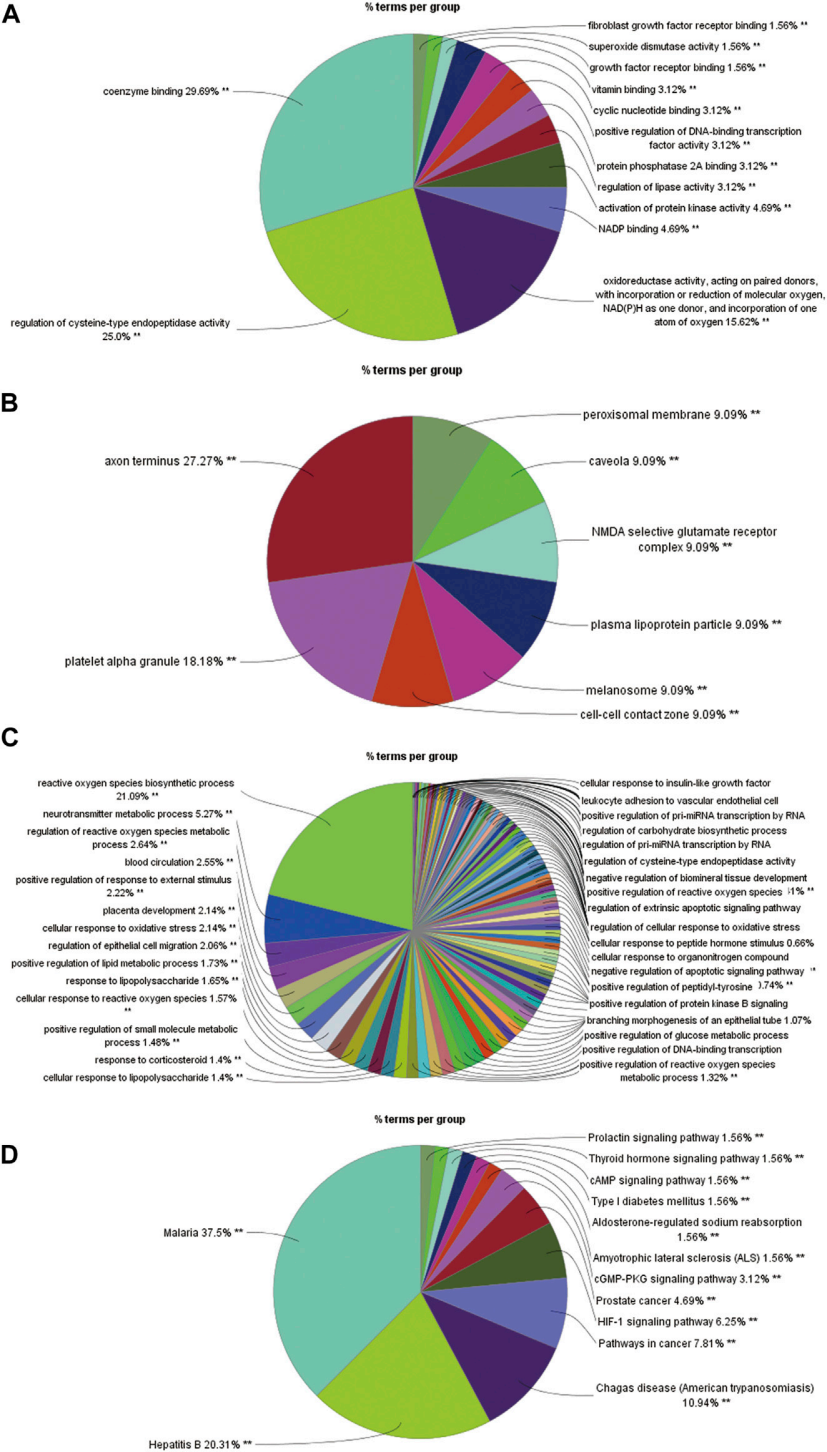
**(A)** Histogram of the results from the molecular functional enrichment analysis (*p* ≤ 0.01). **(B)** Pie chart of the results from the cell component enrichment analysis (*p* ≤ 0.05). **(C)** Pie chart of the results from the biological process enrichment analysis (*p* ≤ 0.01). **(D)** Diagram of the results from the pathway enrichment analysis (*p* ≤ 0.01).

Using DAVID, a total of 112 intersecting genes were annotated with respect to biomolecular function. The acquired target information was used for GO annotation analysis and KEGG pathway analysis. The GO annotation analysis was divided into three categories: molecular functions, cellular components and biological processes. The analysis of the molecular function category revealed that the intersection genes mainly participate in the coenzyme binding function, and the related genes include AADAT, ADORA1 and ADRB2 (Figure 7A). The intersection genes were mainly distributed in the axon terminus, and various genes, such as ADORA1, CHRM2 and GAD1, are related to this location (Figure 7B). With respect to biological processes, the intersection genes mostly participate in the response to reactive oxygen species, and the related genes include AKT1, BCL2 and CAT (Figure 7C). The KEGG analysis yielded a total of 51 pathways, which included 81 intersecting genes (Figure 7D). The pathways were screened using the criterion *p* ≤ 0.01, and 13 pathways and 73 genes were ultimately obtained. Enrichment information obtained from the KEGG pathway analysis revealed that *R. Scrophulariae* might play a role by regulating the HIF-1 signaling pathway, thyroid hormone signaling pathway, prolactin signaling pathway, cGMP-PKG signaling pathway and cAMP signaling pathway, and the HIF-1 signaling pathway was identified as the main pathway through which *R. Scrophulariae* treats hyperthyroidism.

### Integrated Analysis Involving Metabolomics and Network Pharmacology

**FIGURE 8:**
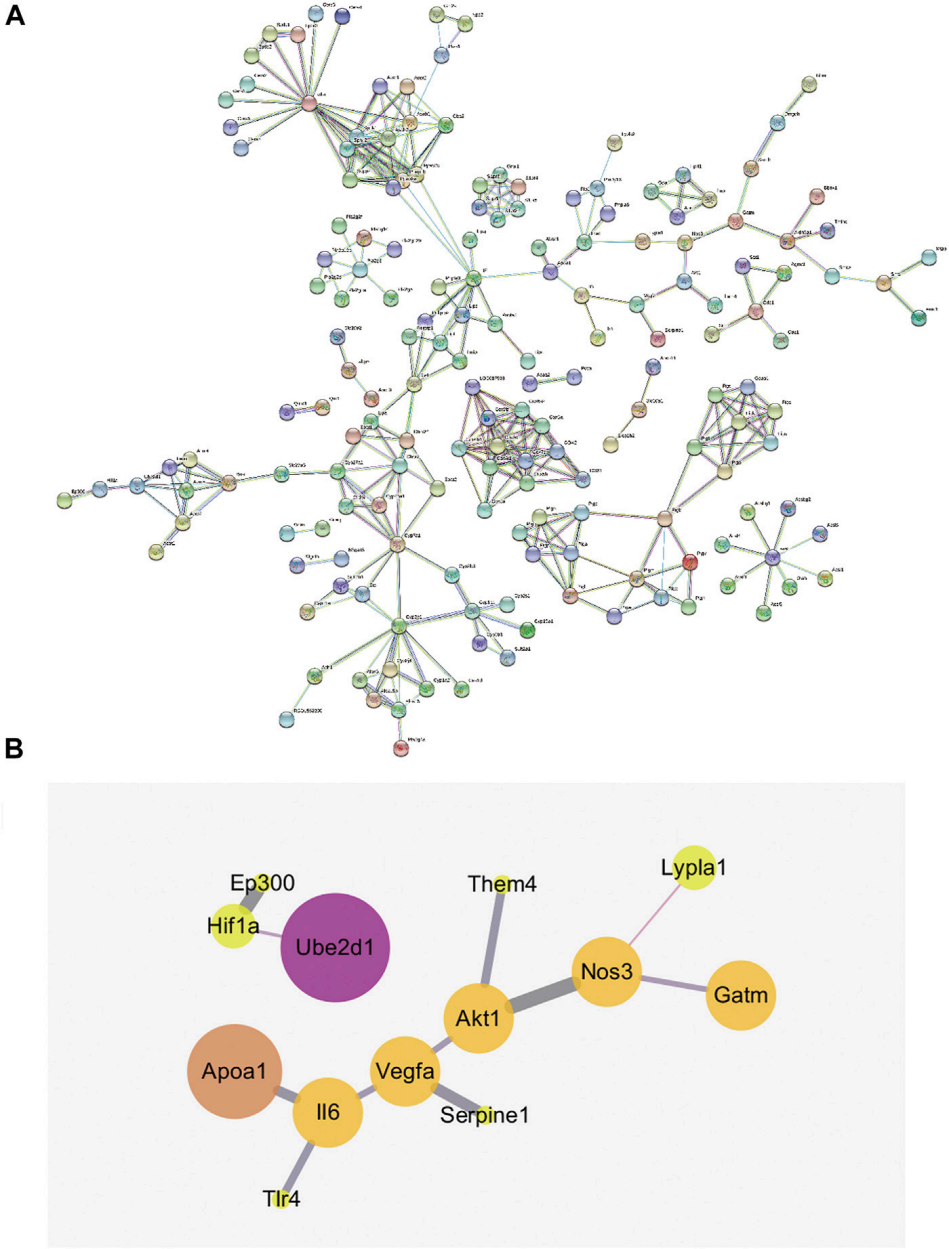
**(A)** Gene interaction network (minimum required interaction score = 0.9). **(B)** Network diagram of local interactions between metabolomics-related genes and HIF-1 signaling pathway-related targets.

The integration of the results from the metabolomics and network pharmacology analyses identified the network of potential biomarkers and HIF-1 signaling pathways that was strongly affected by *R. Scrophulariae* intervention, as shown in Figure 8A. The network contains 251 nodes and 38 interaction relationships and has an average node degree of 2.76 and an average local clustering coefficient of 0.523. A thicker line indicates a stronger interaction. The results from integrated metabolomics and network pharmacology studies revealed that *R. Scrophulariae* might be mainly regulated through the IL6-APOA1-cholesterol pathway, the AKT1-THEM4-palmitic acid pathway,AKT1-THEM4-hexadecanoate (n-C16:0) pathway, the NOS3-LYPLA1-palmitic acid pathway and the NOS3-LYPLA1-hexadecanoate (n-C16:0) pathway to play a role in the treatment of hyperthyroidism, and among these pathways, the IL6-APOA1-cholesterol pathway was the most strongly related to the treatment of hyperthyroidism by *R. Scrophulariae* (Figure 8B).

## Discussion

Patients with hyperthyroidism exhibit hypermetabolism, accelerated breakdown of proteins and fats in the body, and low cholesterol levels (Sabini et al., 2018). In this study, the cholesterol level of hyperthyroidism rats was significantly reduced (*p* ≤ 0.01), and the administration of *R. Scrophulariae* significantly reduced the cholesterol level of the rats (*p* ≤ 0.01), which indicated that *R. Scrophulariae* might treat hyperthyroidism by adjusting the cholesterol level via the lipid metabolism pathway. Cholesterol is involved in the primary bile acid biosynthesis, steroid biosynthesis and steroid hormone biosynthesis pathways in lipid metabolism. However, the primary bile acid biosynthesis, steroid biosynthesis, and steroid hormone biosynthesis pathways are affected by upstream disorders, and these effects are mainly manifested due to an abnormal expression of IL6 in the HIF-1 signaling pathway, which results in disordered lipid metabolism.

Interleukin-6 (IL6) is a cytokine that can be synthesized by a variety of cells. The biological effects of this cytokine are very complex, and its target cells include macrophages, hepatocytes, resting T cells, activated B cells and plasma cells (Karkhur et al., 2019). Hyperthyroidism is considered an autoimmune disease (Lancaster et al., 2019), and the IL6 levels in patients with hyperthyroidism are significantly higher than those in the control group, which indicates that patients with hyperthyroidism exhibit immune dysfunction and that IL6 participates in the pathogenesis of hyperthyroidism. *In vitro* studies have confirmed that the binding of the complex of IL6 and the soluble IL6 receptor (sIL6-R) complex to gp130 induces formation of an IL6 signal transducer, which can transmit the IL6 signal. In the presence of sufficient sIL6-R, IL6 dose-dependently inhibits thyroid function and the release of T3 and T4 (De Filippo et al., 2015; Chen et al., 2016). IL6 is involved in the regulation of thyroid function (Park et al., 2019; Qu et al., 2019).

ApoA1 is the main cholesterol-related gene, and apolipoprotein A-I is the main protein component of high-density lipoprotein in the blood circulation and the main receptor for cholesterol in extrahepatic tissues. This protein plays an important role in the transport and metabolism of cholesterol because it can remove excess lipid deposits on the vascular walls to protect blood vessels (Suematsu et al., 2019).

The content of APOA1 in the serum of pregnant women with hyperthyroidism at the early stage of pregnancy does not significantly differ from that found in normal pregnant women. The serum APOA1 content observed in pregnant women with hyperthyroidism at the middle and late stages of pregnancy is significantly lower than that found in normal pregnant women. The possible mechanism for this difference is that the liver is the main site for the synthesis of APOA1, and thyroid hormones can promote APOA1 gene transcription, but long-term thyroid hormone stimulation would inhibit the synthesis of APOA1 mRNA. This negative feedback regulation mechanism might lead to continuous increases in the APOA1 levels in the serum of normal pregnant women but would result in no significant changes in the levels found in pregnant women with hyperthyroidism; as a result, pregnant women with hyperthyroidism have a lower serum APOA1 content than normal pregnant women at the second and third trimesters (Einbinder et al., 2018; Kelly et al., 2019; Li et al., 2019; Retnakaran et al., 2019). Therefore, *R. Scrophulariae* might treat hyperthyroidism by upregulating the APOA1 and cholesterol levels while simultaneously downregulating the IL6 levels and regulating the IL6-APOA1-cholesterol pathway via the HIF signaling pathway.

## Conclusion

*R.* Scrophulariaceae exerts a callback effect on the 13 metabolic pathways affected by Euthyrox:unsaturated fatty acid biosynthesis, primary bile acid biosynthesis, sphingolipid metabolism, β-alanine metabolism, glutathione metabolism, arginine and proline metabolism, linoleic acid metabolism, mitochondrial fatty acid elongation, fatty acid metabolism, and fatty acid biosynthesis, steroid biosynthesis, steroid hormone biosynthesis, and purine metabolism. The *R. Scrophulariae*-active ingredient-target-disease-related network was constructed using network pharmacology approaches. A total of 73 interaction targets and 13 related pathways were found, and among these pathways, the HIF-1 signaling pathway was the main pathway associated with the treatment of hyperthyroidism by *R. Scrophulariae*. The integration of metabolomics and network pharmacology revealed that *R. Scrophulariae* might mainly regulate the IL6-APOA1-cholesterol pathway, AKT1-THEM4-palmitic acid pathway, AKT1-THEM4-hexadecanoate (n-C16:0) pathway, NOS3-LYPLA1-palmitic acid pathway and NOS3-LYPLA1-hexadecanoate (n-C16:0) pathway to play a role in the treatment of hyperthyroidism. This study of hyperthyroidism disease using an approach that integrates metabolomics with network pharmacology provides new ideas for further research on the pharmacological mechanism of *R. Scrophulariae* and its material basis.

## Data Availability

The original contributions presented in the study are included in the article/Supplementary Material, further inquiries can be directed to the corresponding authors.

## Abbreviations

TCM, Traditional Chinese Medicine; BPI, base peak intensity; PLS-DA, partial least squares discriminate analysis; OPLS-DA, orthogonal partial least squares discriminate analysis; VIP, variable importance in the projection; OB, oral bioavailability; DL, drug-likeness; PPI, protein-protein interaction; QC, quality control; PCA, principal component analysis; IL6, interleukin-6; sIL6-R, soluble IL6 receptor.
